# Bim and VDAC1 are hierarchically essential for mitochondrial ATF2 mediated cell death

**DOI:** 10.1186/s12935-015-0188-y

**Published:** 2015-03-30

**Authors:** Zhaoyun Liu, Qianfu Luo, Chunbao Guo

**Affiliations:** Laboratory of Surgery, Children’s Hospital of Chongqing Medical University, 136 Zhongshan 2nd Rd, Chongqing, 400014 P. R. China; Ministry of Education Key Laboratory of Child Development and Disorders, Chongqing, P. R. China

**Keywords:** Mitochondria, ATF2, Bim, VDAC1, Apoptosis

## Abstract

**Background:**

ATF2 mediated cytochrome c release is the formation of a channel with some unknown factors larger than that of the individual proteins. BHS-only proteins (BH3s), such as Bim, could induce BAX and VDAC, forming a new channel. According to this facts, we can speculated that there is possible signal relationship with BH3s and ATF2, which is associated with mitochondrial-based death programs.

**Methods:**

The growth inhibitory effects of mitochondrial ATF2 were tested in cancer cell lines B16F10, A549, EG7, and LL2. Apoptosis was measured by flow cytometry. The effects of ATF2 and levels of apoptosis regulatory proteins were measured by Western blotting. The interaction of proteins were evaluated by immunoprecipitation analysis. The in vivo antitumor activity of mitochondrial ATF2 were tested in xenograft B16F10 models.

**Results:**

Genotoxic stress enabled mitochondrial ATF2 accumulation, perturbing the HK1-VDAC1 complex, increasing mitochondrial permeability, and promoting apoptosis. ATF2 inhibition strongly reduced the conformational activation of Bim, suggesting that Bim acts downstream of ATF2. Although Bim downregulation had no effect on ATF2 activation, Bim knockdown abolished VDAC1 activation; the failure of VDAC1 activation in Bim-depleted cells could be reversed by the BH3-only protein mimic ABT-737. We also demonstrate that silencing of ATF2 in B16F10 cells increases both the incidence and prevalence of tumor xenografts in vivo, whereas stably mitochondrial ATF2 transfection inhibited B16F10 tumor xenografts growth.

**Conclusions:**

Altogether, these results show that ATF2 is a component of the apoptosis machinery that involves a hierarchical contribution of ATF2, Bim, and VDAC1. Our data offer new insight into the mechanism of mitochondrial ATF2 in mitochondrial apoptosis.

## Introduction

As one member of activator protein-1 (AP-1) transcription factor, a growing number of transcription dependent and independent functions have been described, while attesting to its capacity to regulate diverse and often opposing functions, which implicated some of the most challenging therapeutic targets [1,2]. Growing evidence indicates that a cytoplasmic localization of activating transcription factor 2 (ATF2) is associated with cell death in disease or cellular stress states [3]. Following ionizing irradiation with prostate cancer cells, ATF2 had been observed to accumulate in the cytoplasm [4]. Furthermore, the forced expression of cytoplasmic ATF2 peptides induces melanoma cell death and thereby reduces the transcriptional activity of endogenous ATF2 [5]. Further studies have shown that genotoxic stress induces translocation of nuclear ATF2 to the cytoplasm, which coincides with the reduced transcriptional activity of ATF2 [6,7].

Because ATF2 localizes at the mitochondrial outer membrane result in reduced mitochondrial membrane potential, with concomitant leakage from the mitochondria, hallmarks of mitochondrial-dependent cell death. Hence, ATF2 recruitment to the mitochondria is associated with its tumor suppressor activities. Despite the fact that functional studies of ATF2 provide no compelling reason for decreasing HK1 bind to VDAC1 [8,9], possible mechanism might be formation of VDAC channel with some unknown factors larger than that of the individual proteins to mediate cytochrome c release. ATF2 also reported play an important role in the conformation and thus activation of Bax [1]. Conformational changes and homo-oligomerization are two critical events associated with the activation of BAX/BAK by BH3s and VDAC1. However, the underlying mechanisms remain unsettled. It is especially complex for BAX due to its change in the subcellular localization during apoptosis. An understanding of the precise mechanisms underlying its function at the mitochondria, could provide a platform for identifying small molecues that affect the involved signaling. Similarly, it will be important to determine its mitochondrial function and their possible relationship to the Bcl-2 family protein signaling that is associated with mitochondrial-based death programs [10,11]. Indeed, evidence involved in activator BHS-only proteins (BH3s), such as Bim, has been presented, which could induce BAX and VDAC reconstituted into liposomes, forming a new channel at the MOM [12-14]. According to this facts, we can speculated that there is possible signal relationship with BH3s and ATF2, which is related to mitochondrial involved apoptosis.

Here, we investigate the role of ATF2 in mitochondrial apoptosis and the mechanisms underlying the interaction of ATF2 with other proapoptosis proteins. Our work revealed that, at the membrane of mitochondrial, the hierarchical involvement of Bim, VDAC1, and ATF2 break the hexokinase-1 (HK1) and voltage-dependent anion channel-1 (VDAC1) complexes, thereby enhancing mitochondrial permeability and releasing cytochrome *c*. The exploration of the mechanisms for ATF2 mitochondrial localization offers a theoretical basis to guide cancer cells to apoptosis.

## Methods

### Cell culture, antibodies, regents, and drugs

The tumor cell lines B16F10, A549, EG7, and LL2 were selected for this research. These cells were gifts of Prof. Hongbo Luo, Harvard University, and were maintained in RPMI 1640 (Gibco, Gaithersburg, USA) or DMEM (Life Technologies, CA, USA), each supplemented with 10% fetal bovine serum and penicillin G sodium and streptomycin sulfate (100 units/ml) in a humidified atmosphere of 5% CO2 and 95% air. ABT-737 (Selleckchem, Souffelweyersheim, France) used at 1 *μ*M final concentration. MitoTracker Red CMXRos (M7512) and leptomycin B (LMB) (sc-358688) were purchased from Invitrogen (Eugene, OR, USA) and Santa Cruz Biotechnology (Santa Cruz, CA, USA), respectively. All antibodies including ATF2(C-19, sc-187), p-ATF2(F-1, sc-8398), COX4 (D-20, sc-69359), Bim (N-20, sc-8265), PUMAα (N-19, sc-19187), Bax (P-19, sc-526), cytochrome c (C-20, sc-8385), VDAC1 (B-6, sc-390996), Mcl-1 (S-19, sc-819), β-Actin (C4, sc-47778) were purchased from Santa Cruz Biotechnology. The monoclonal anti-HK1(C35C4, 2024) antibody was obtained from Cell Signaling Technologies (MA, USA). Paclitaxel ((PTX, 33069-62-4) was obtained from Beijing Zhongshuo Pharmaceutical T & D Co., Ltd (Beijing, China). Staurosporine (STS), cisplatin, 4,4′-diisothiocyanostilbene-2,2′-disulfonic acid (DIDS), carbonyl cyanide m-chlorophenylhydrazone (CCCP), and propidium iodide were purchased from Sigma (St. Louis, MO, USA). The annexin V-FITC kit (K201-100) was purchased from Biovision. PTX was purchased from Calbiochem (La Jolla, CA) and dissolved in 100% dimethyl sulfoxide to produce a stock solution of 1.0 mM.

### RNA interference

Cells were transfected at ~70-90% confluence (approximately 1 × 10^5^ cells/ml density). ATF2-specific shRNA clones (ID: TRCN0000013713, Sigma, USA) were obtained from Open Biosystems (catalog no. RHS4533). Lentiviral particles packaged with ATF2 shRNA or scrambled shRNA (control) were generated and spin infected into the target cells in the presence of 10 mg/ml polybrene (Sigma, USA) [15]. The transfection of a synthetic siRNA (25 nM) for VDAC1 was performed with Lipofectamine 2000 (Invitrogen, Carlsbad, CA, USA) according to the manufacturer’s instructions. The sense sequence of the double-stranded siVDAC1 siRNA was 5′ AGUGACGGGCAGUCUGGAATT 3′. A scrambled siRNA, which served as a negative control, was purchased from GenePharma (Shanghai, China). HK1 siRNAs (ID: 1599) were obtained from Ambion (USA). The same siRNA reagents were added to the medium at 24 hours post-transfection for 24 hours. The western blot analysis was used to evaluate gene silencing effects. The appropriate controls were included during the entire siRNA knockdown process, confirming the specificity of the siRNA. For the construction of tetracycline-regulated gene expression vectors expressing ATF2^T52A^ mutants (mitochondria localization), DNA was amplified by PCR using pEF-HA-ATF2 (WT, T52A mutants), a gift of Ze’ev A. Ronai (Sanford-Burnham Medical Research Institute, La Jolla, CA 92037, USA), and subcloned into pTHE, resulting in pTHE-WT, ATF2^T52A^, which were transfected into B16F10 and screened with tetracycline.

### Cell viability determination (MTT Assay)

Subconfluent monolayers of the B16 cell line were established in 96-well plates. After overnight incubation, the cells were exposed to different treatments in a medium containing 0.5% fetal bovine serum for 72 hours; 10-μl aliquots of 3-(4,5-dimethylthiazol-2-yl) 2,5-diphenyl-tetrazolium bromide (MTT) solution (10 mg/mL in PBS) were added, followed by 100 μL of 10% sodium dodecylsulfate (SDS) to dissolve the formazan crystals formed. The absorption of the samples was determined using an ELISA reader (Anthos Mikrosysteme GmBH, Germany) at a wavelength of 570 nm. A standard optical density of the untreated control cells was considered at 100% viability. Survival was evaluated by the absorbance of the treated cells normalized to the controls.

### Confocal immunofluorescence assays

Cells from different treatment groups were incubated with MitoTracker Red (25 nM) for 15 minutes, fixed, permeabilized, and stained with antibodies for the detection of ATF2 (20 F1). The primary antibodies were revealed using either goat anti-rabbit or anti-mouse IgG conjugated to Alexa 488 (green) (1:500, diluted in blocking solution) from Molecular Probes-Invitrogen. After 1 hours of incubation, the slides were mounted, and the stained cells were analyzed using a confocal microscope (Leica Microsystems Heidelberg GmbH, Heidelberg, Germany).

### Apoptosis assay by annexin V/propidium iodide staining

At various time points, control and treated cells were collected following treatment and subjected to apoptosis measurement using the annexin V/propidium iodide (PI) detection kit (R&D Systems) according to the manufacturer’s instructions. A total of 10,000 cells (within whole-cell gates) per replica (3 independent experiments) were subjected to a flow cytometric analysis to evaluate the green fluorescence of annexin V and the red fluorescence of DNA-bound PI. All the data were analyzed with FlowJo software (TreeStar, OR).

### Cytochrome c release assay

Isolated tumor cells (5 × 10^7^) were collected and assayed with the Cytochrome c Apoptosis Assay Kit (Cat. #K257-100, Biovision, CA, USA). Briefly, the cells were homogenized with the cytosol extraction buffer provided in the kit and then centrifuged at 700 × g for 10 minutes at 4°C to remove the debris. The supernatant was then centrifuged at 10,000 × g for 30 minutes at 4°C; the pellet contained the mitochondrial fraction, and the supernatant was collected as the cytosolic fraction. These fractions were analyzed for cytochrome *c* by western blotting using the cytochrome *c* antibody provided in the kit.

### Immunoprecipitation and analysis of protein expression

Cells, transfected as indicated, were lysed in the buffer for 45 min. Lysate aliquots of equal concentration were then incubated overnight with 2 μg of anti-ATF2, −VDC1, −Bim, and -Puma antibodies in an overhead rotator, followed by 20 μl protein G-Sepharose beads (Amersham Pharmacia Biotech, Uppsala, Sweden) for 2 h. The immunoprecipitated proteins were incubated at 70°C for 15 min and analyzed by immunoblotting with conformation-specific primary antibodies against ATF2, VDC1, Bim, Puma, HK1, and VDAC1 (Cell Signaling Technology). β-actin (Chemicon International, Temecula, CA, USA) was performed as loading control.

### Cell fractionation

Fractions of cytoplasm nuclear, and mitochondria were separated using a commercial Qproteome mitochondria extraction kit and a Qproteome nucleus extraction kit (Qiagen, Toronto, ON, Canada). Briefly, cells were firstly lysed and centrifuged for 5 min at 1000 × g to remove unbroken cells and nuclei. The supernatant was separated from the pellet and centrifuged at 2,200 × g for 20 min at 4°C to pellet the mitochondria-enriched heavy membrane fraction. The resulting supernatants were combined and further centrifuged at 4°C at 12,000 × g for 30 min at 4°C to obtain the cytoplasmic fraction. An immunoblot analysis was performed as described below.

### Western blot analysis

Cells from different treatment groups were lysed using a protein extraction buffer. Total proteins (10 μg) were separated by SDS-PAGE and transferred to nylon membranes (Shanghai Sangon Biotech, Shanghai, China). The blots were hybridized with antibodies indicated above. The secondary antibody, horseradish peroxidase-coupled immunoglobulin (Jingmei Biotech Co., Ltd. Shenzhen, China), was then inculated for 1 h. β-actin (Sigma) was used as loading control. All critical blots and immunoprecipitation experiments were repeated at least three times.

### Mitochondrial membrane potential detection

Cells were treated and resuspended in serum-free medium at a concentration of 1 million cells/ml. Each sample was added 5 μl of JC-1 dye (200 μM) for incubation at 37°C, 30 min. The samples were measured by flow cytometry, with 10,000 events collecting. Results were also observed under fluorescence microscopy.

### Tumor implantation procedure

C57BL/6 female (8–10 weeks old) mice were purchased from Chongqing Medical University Animal Center (Chongqing, China). All animal experiments were performed with the approval of the Animal Institute Committee. B16F10 cells stably transfected with ATF2 shRNA, ATF2^T52A^ or with empty vector (1.0 × 10^6^/0.1 ml) were injected subcutaneously. The tumor sizes were evaluated using calipers every 2 to 3 days, and the tumor volumes were calculated using the formula: volume = (a^2^ × b)/2 (a, the short tumor length; b,the long tumor length). In one arm of the experiment, nonnecrotic, single-cell suspensions from tumor tissue were prepared for FACS staining of annexin V/propidium iodide. A portion of the freshly isolated tumor tissue was subjected to a western blotting assay and real-time PCR analysis, as described in the results section.

### Statistical analysis

Data are expressed as means ± standard errors of the mean (SEM). Unless indicated otherwise, comparisons were determined using the Student’s t test and one-way ANOVA. P < 0.05 were considered as significance difference.

## Results

### ATF2 mitochondrial localization is critical for genotoxic-induced apoptosis

**Figure 1:**
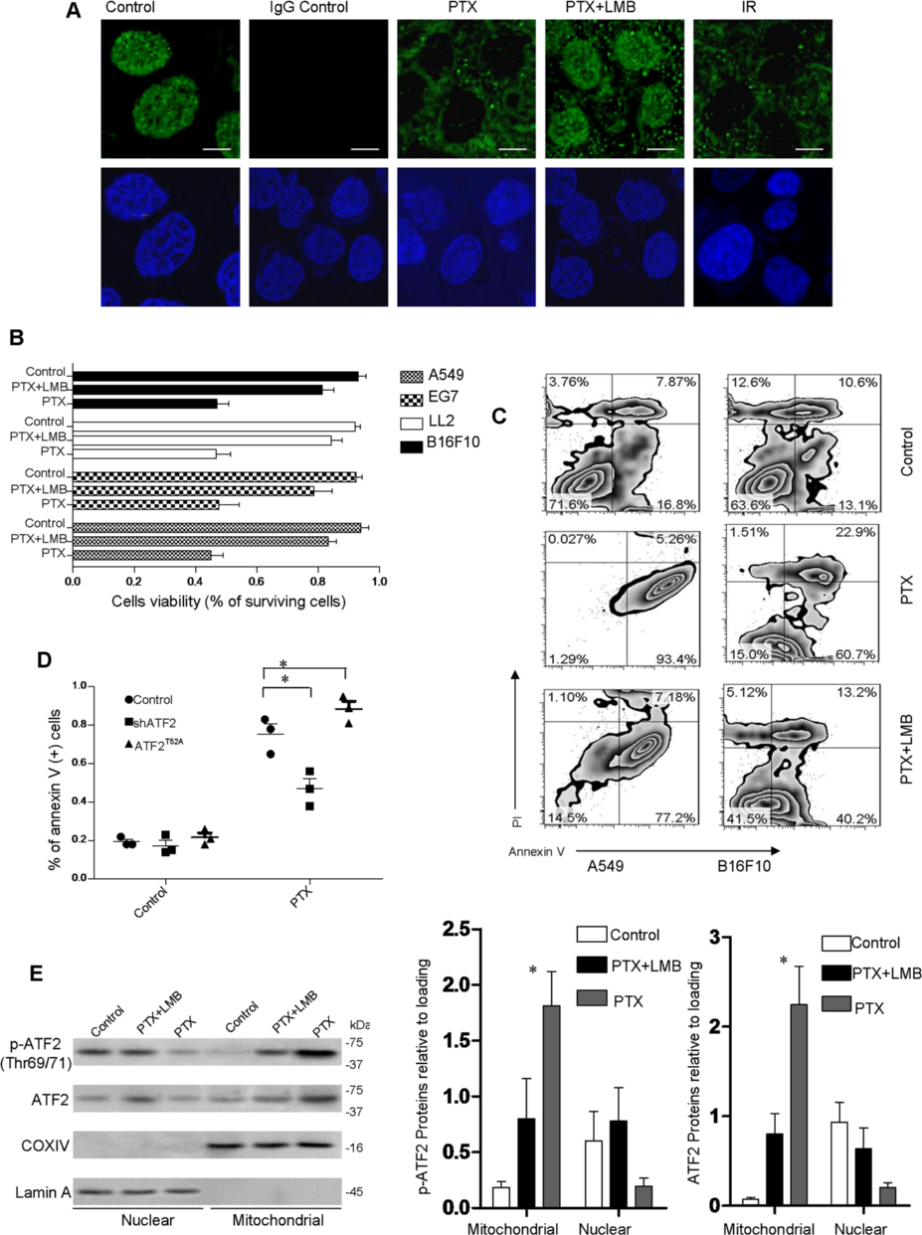
**Role of ATF2 in PTX-induced apoptosis in various tumor cell lines. (A)** Control, 100 nM PTX-treated (12 hours treatment), and ionizing radiation-treated (IR, 5 Gy) cells were permeabilized and stained for ATF2. The nuclear localization of ATF2 is indicated by costaining with DAPI (Vector Laboratories, UK). Scale bar: 20 μm. **(B)** The inhibition of cell viability with PTX or leptomycin B (LMB) and PTX cotreatment was assessed by the (4,5-dimethylthiazol-2-yl)2,5-diphenyl-tetrazolium bromide assay. Data are means ± s.e.m and represent the means from three different experiments. **(C)** Diagrams of flow cytometry showing the results from the detection of apoptosis with the cells treated as indicated above. **(D)** Shown is a diagram of three similar FACS apoptosis assay using Annexin V/PI staining for B16F10 cells stably transfected with scrambled or ATF2 shRNA incubation in the absence or presence of PTX for 12 hours. Statistical analysis of apoptosis in the different treatments was performed by ANOVA and t-tests; *P < 0.01. Data are means ± s.e.m (n = 4). **(E)** Different fractionations purified from the indicated cells were subjected to western blotting with COXIV and β-Actin as the loading control. Representative figures of multiple experiments are shown. The morphometry of p-ATF2 and ATF2 are indicated at right. The columns provide average values of at least 3 independent experiments performed in triplicate; the bars, SE. *P < 0.01 compared with the corresponding control.

To test the contribution of mitochondrial ATF2 to apoptosis, we measured the cytotoxic effect of genotoxic insults on several cancer cell lines by a cell viability analysis while measuring the ATF2 mitochondria accumulation within these cells. As shown in Figure 1A, mitochondrial accumulation was induced in the mouse tumor cell line B16F10 as early as 4 hours after paclitaxel (PTX) treatment, whereas leptomycin B (LMB), the nuclear export inhibitor, prevented the ATF2 mitochondrial localization. PTX also resulted in 20% growth inhibition. Similar effects were observed in other cell types, including A549, EG7, and LL2 (Figure 1B). This reduced viability was due to the activation of an apoptotic program, as revealed by a flow cytometric analysis The induction of apoptosis can also be prevented by pretreatment with LMB (Figure 1C).

We next compared the contribution of ATF2 to apoptosis in cells using ATF2 shRNA transfection. The B16F10-shATF2 cells exhibited more resistance to PTX-induced apoptosis than the control phenotype (P <0.01) (Figure 1D). To assess the effect of elevated mitochondrial ATF2 expression in B16F10 cells, cells were transfected with the ATF2^T52A^ expression vector, which preferentially localizes to the mitochondria [3]. The exposure of cells expressing mitochondrial ATF2 to PTX exhibit significantly higher Annexin-V uptake (i.e., cell death) compared to the cells expressing the control vector (Figure 1D). We also found that genotoxic stimuli could also induce a high level phosphorylated (p-ATF2, at Thr69/71), the active form of mitochondrial ATF2 (Figure 1E), consistent with the cell viability performance after challenge with genotoxic stimuli.

### Bim and VDAC1 are required for ATF2-mediated cell apoptosis

**Figure 2:**
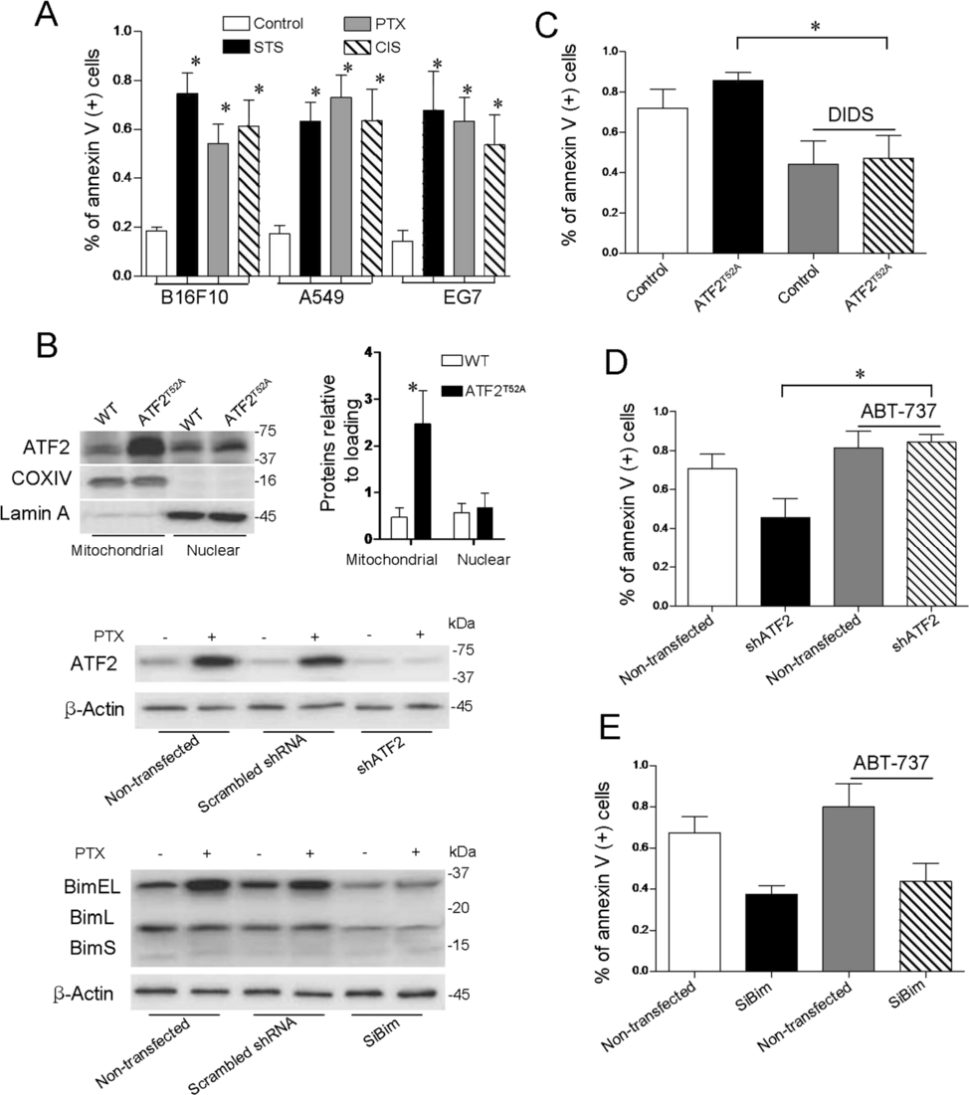
**Bim and VDAC1 are involved in ATF2-associated apoptotic induction. (A)** Different cancer cell lines were subjected to mitochondria-mediated apoptosis with PTX, STS (1.25 μM, 5 hours), and cisplatin (50 μM, 30 hours) and apoptosis analysis by Annexin V/PI staining. Data are means ± s.e.m and represent three different experiments. *P < 0.01, ANOVA, versus Control. **(B)** The B16F10 cells were transfected with siBim (low panel), shATF2 (middle panel) and ATF2^T52A^ (up panel), and whole-cell lysate for siBim and shATF2 or fractionated to Nuclear and mitochondrial fractions for ATF2^T52A^ were subject to immunoblot analysis with indicated antibodies. β-actin loaded as control. The morphometry of ATF2 are indicated at right. The columns provide average values of at least 3 independent experiments performed in triplicate; the bars, SE. *P < 0.01 compared with the corresponding control. **(C)** B16F10 cells, transfected with ATF2^T52A^ or not were treated with PTX (100 nM, 12 hours), pretreatnent with or without DIDS (100 μM, 1 h). Cells were then subjected to measurement of apoptosis by Annexin V/PI staining. Columns, mean of three individual experiments; bars, s.e.m. *P < 0.01, ANOVA. **(D)** Cells expressing shATF2 were exposed in the absence or presence of ABT-737 (1*μ*M, 24 h) and subjected to mitochondria-mediated apoptosis induction by PTX. Cells were then subjected to measurement of apoptosis by Annexin V/PI staining. Columns, mean of three individual experiments; bars, s.e.m. *P < 0.01, ANOVA. **(E)** Cells expressing siBim were exposed in the absence or presence of ABT-737 and then subjected to mitochondria-mediated apoptosis induction by PTX. FACS analyses of apoptotic cell death were performed using Annexin V/PI staining. Columns, average values of three independent experiments; bars, s.e.m. *P < 0.01, ANOVA.

As Bim and VDAC1 are reported to induce mitochondrial apoptosis, we explored the roles of these proteins in ATF2-related cell death. As with the cells possessing a specific level of ATF2, all the cell types were sensitive to apoptosis induction by apoptosis-inducing agents, such as PTX, staurosporine (STS), and cisplatin, which act through the activation of ATF2 (Figure 2A).

Approximately 80% of the ATF2^T52A^-expressing cells underwent apoptosis upon exposure to PTX, which was significant increased compared with WT control (Figure 2B). the efficience of ATF2^T52A^ transduction was proved in Figure 2B. We next inhibited VDAC1 function in ATF2^T52A^-expressing cells using a chemical inhibitor of VDAC1, 4,4′-diisothiocyanostilbene-2,2′-disulfonic acid (DIDS). As expected, apoptosis induction by ATF2 was significantly prevented upon VDAC1 depletion (Figure 2C).

In contrast to ATF2^T52A^-expressing cells, the ATF2-depleted cell exhibited resistance to apoptosis induction by PTX (Figure 2D). We also investigated the possibility of restoring the sensitivity of ATF2-depleted cells to PTX with ABT-737, the pharmacological mimetic of Bcl-2 homology domain 3 (BH3), which is thought to act by displacing Bim from Bcl-2. We firstly separately confirmed the efficiencies of the knockdowns (ATF2 and Bim) by Western blot analysis (Figure 2B). PTX treatment alone resulted in lower levels of annexin-V in the ATF2-depleted cells. Conversely, ABT-737 sensitized the ATF2-depleted cells to PTX induced apoptosis as efficiently as the control cells (Figure 2D). Notably, the siBim conferred resistance to treatment, which completely abrogated the effects of ABT-737 (Figure 2E).

### ATF2 competes with HK1 to bind VDAC1

**Figure 3:**
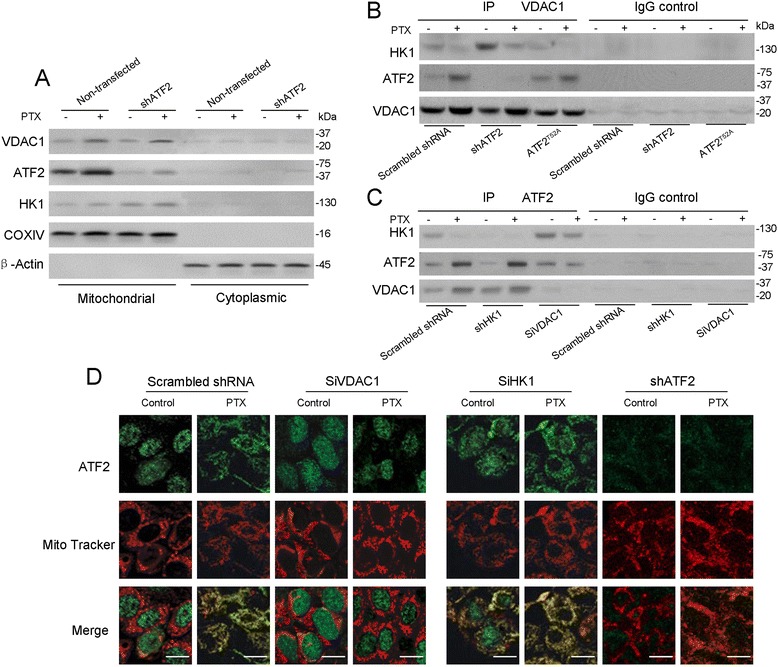
**The interaction of VDAC1 and HK1 with ATF2 in response to apoptosis induction. (A)** B16F10 cells stably transfected with scrambled or ATF2 shRNA were treated with PTX or PBS. Mitochondrial and Cytoplasmic extracts were subjected to an immunoblot analysis with the indicated antibodies. The positions of the molecular weight markers (in kDa) are noted on the right. **(B)** B16F10 cells stably transfected with ATF2 shRNA or ATF2^T52A^ were crosslinked and lysed. VDAC1 was immunoprecipitated and subjected to an immunoblot analysis with the indicated antibodies. **(C)** B16F10 cells stably transfected with HK1 shRNA or VDAC1 shRNA were subjected to immunoprecipitated for ATF2 and then measured with immunoblot analysis using the indicated antibodies. **(D)** Cells stably transfected with HK1 shRNA, ATF2 shRNA, and VDAC1 shRNA were immunofluorescently stained with MitoTracker and anti-ATF2 antibodies after exposure with or without PTX and visualized using confocal microscopy (Olympus 1X81) (Scale bar: 20 μm).

As VDAC1 played an critical role in mitochondrial permeabilizing pores, we next addressed whether the mitochondrial translocation of ATF2 perturbs VDAC1 in these pores in B16F10 cells. PTX was predictably observed to significantly increase the protein level of ATF2, bur no influence the HK1 and VDAC1 level in the mitochondria of B16F10 cells after 4 h of treatment (Figure 3A). To identify the interactions of ATF2 with VDAC1 or HK1, endogenous VDAC1 were immunoprecipitated and bloted with ATF2 in B16F10 cell lysates prior to and after PTX stress. ATF2 and VDAC1 interaction wwere clearly observed after PTX treatment (Figure 3B), whereas the interaction between HK1 and VDAC1 was disturbed by endogenous ATF2 accumulation (Figure 3B) and ATF2 depletion promoted the association of HK1 and VDAC1.

Because we observed that the level of VDAC1 was elevated in the ATF2-overexpressing B16F10 cells, we next assessed whether VDAC1 depletion would affect the mitochondrial accumulation of ATF2. As expected, we observed that the protein levels of ATF2 were significant altered after VDAC1 knockdown (Figure 3C). At the same time VDAC1 depletion seems promoted HK1 association with ATF2 under basal conditions and following PTX treatment (Figure 3C). We then observed that more mitochondrial ATF2 accumulation in the HK1 depletion cells even exposure with PTX (Figure 3C), suggesting that ATF2 accumulation was affected by HK1 reduction, and the VDAC1 protein level immunoprecipitated from ATF2 was significant improved, suggesting HK1 depletion facilitated the association of mitochondrial ATF2 with VDAC1 (Figure 3C). These results suggest that HK1 and ATF2 most likely compete for binding to VDAC1 on the outer mitochondrial membrane.

We further demonstrated the VDAC1 and ATF2 interaction by cellular distribution using confocal fluorescence microscopy. Under basal conditions, ATF2 is located in the nucleus and cytoplasm. In the PTX-treated cells, ATF2 fluorescence was attenuated with the nuclear localization of ATF2 and promoted with mitochondrial localization, as punctuate, MitoTracker-labeled mitochondrial structures, suggesting that PTX treatment enabled ATF2 nuclear export and mitochondrial localization (Figure 3D). MitoTracker Red is highly sensitive to mitochondrial membrane potential (mmp) and thus decreased labeling exhibited decreased mmp in PTX treated cells. Consistently, the cell expressing the VDCA1 siRNAi showed decreased mitochondrial ATF2 localization, even in the presence of PTX, as detected by mitochondrial fractionation and diffuse staining throughout the cytosol (Figure 3D) in comparison with the the empty vector expressing cells, suggesting that the ATF2 mitochondrial binding required VDAC1. Additionally, HK1 depletion enhanced the ATF2 translocation in mitochondrial following PTX stress (Figure 3D).

### Hierarchical relationship between ATF2, Bim, and VDAC1

Previous studies have shown that VDAC1 promotes the mitochondrial accumulation of BAX to trigger the subsequent cytochrome *c* release, however, Bim, a mitochondrial membrane protein, is also essential for the activation of BAX and release of cytochrome *c* (14). Accordingly, we examined whether ATF2 would affect the activation status of Bim, thereby promoting the interaction between BAX and VDAC1.

**Figure 4:**
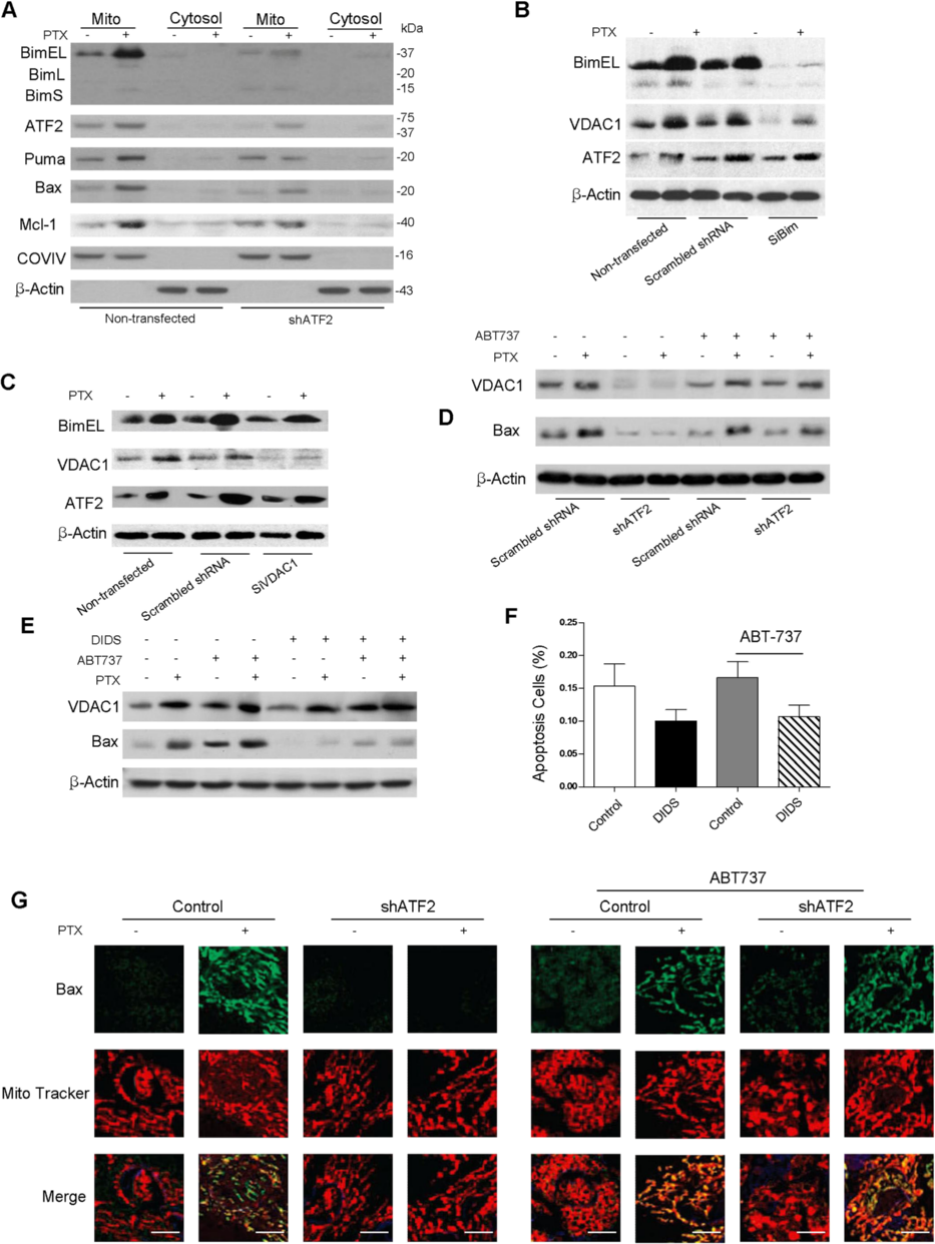
**ATF2 affects the PTX-elicited activation of Bim and Puma. (A)** B16F10 cells treated with PTX were examined for the subcellular localization of proteins by western blotting, with the protein weights indicated (in kDa). COXIV was shown as a loading control of mitochondrial sample; β-Actin was shown as an loading control of cytosolic fraction. B16F10 cells stably transfected with scrambled or Bim SiRNA **(B)**, VDAC1 SiRNA **(C)** and ATF2 shRNA **(D)** were subjected PTX treatment and then subject to immunoblot analysis with the indicated antibodies. B16F10 cells were preincubated with or without DIDS (100 μM, for 1.5 h), followed by ABT737 (1*μ*M, 24 h) and PTX treatment (100 nM, 12 h). Cells were subjected to immunoblot analysis with the indicated antibodies **(E)** and FACS apoptosis analyses Annexin V/PI staining **(F)**, *P < 0.01, ANOVA. Data are means ± s.e.m. (n = 5). **(G)** B16F10 cells stably transfected with scrambled or ATF2 shRNA were incubated with ABT-737 alone or in conjunction with PTX. The cells were immunofluorescently stained with the indicated antibodies and visualized using confocal microscopy (Olympus 1X81) (Scale bar: 20 μm).

Consistently, we found that PTX treatment resulted in Bim and Puma mitochondrial expression, as revealed by a fractionation analysis of immunoblotting (Figure 4A). A 62% induction in ATF2 levels caused the efficient activation of Bim, whereas ATF2 depletion reduced the activation of Bim and Puma to minimal levels (Figure 4A). Next, we performed immunoprecipitation assays to directly measure the interaction between Bim and ATF2 or VDAC1 in B16F10 cell lysates prior to and after PTX treatment. We did not find a direct binding between Bim and ATF2 or Bim and VDAC1; however, when Bim was silenced with siRNA, we observed a significant inhibition of Bim but no altered ATF2 activation (Figure 4B). These results suggest that ATF2 interrupts PTX involved signaling at the upstream of Bim level. Furthermore, Bim depletion strongly inhibited the PTX-induced VDAC activation, whereas VDAC1 depletion had no effect on Bim induction (Figure 4C). Taken together, we postulate that Bim might take effect at the upstream of VDAC1 and downstream of ATF2.

We next explored the possibility of restoring the ATF2-VDAC pathway by ABT-737. We found that PTX alone failed to activate BAX in ATF2-depleted cells, whereas ABT-737 could rescue the inhibition of BAX translocation to mitochondria by an ATF2-targeting shRNA (Figure 4D). A fractionation analysis revealed that the PTX and ABT-737 combination efficiently elicit BAX activation in ATF2-targeting shRNA cells (Figure 4D), but ABT-737 could not rescue the inhibition of BAX translocation by VDAC inhibition (DIDS) (Figure 4E), which were further verified in cellular distribution using confocal fluorescence staining (Figure 4G). We also found that VDAC1 was activated by ABT-737 in cells that were apt to apoptosis, whereas VDAC1 inhibition by DIDS induced a large extent or complete resistance to PTX treatment even in the presence of ABT737, which was consist with the finding that VDAC1 was activated by ABT-737 in cells that were also sensitive to PTX (Figure 4F).

Notably, Bim depeltion completely abrogated the effects of ABT-737, preventing it from regaining the sensitive of ATF2-depleted cells to PTX (Figure 2D). Altogether, these results indicate that ATF2 sensitizes cells to apoptosis by promoting Bim and hence to directly facilitate VDAC1 activation. Overall, our study reveals an essential axis of activator ATF2 and Bim and Puma in activating the mitochondrial death program.

### ATF2 localization at the mitochondria occurs essential in promoting cytochrome *c* release

**Figure 5:**
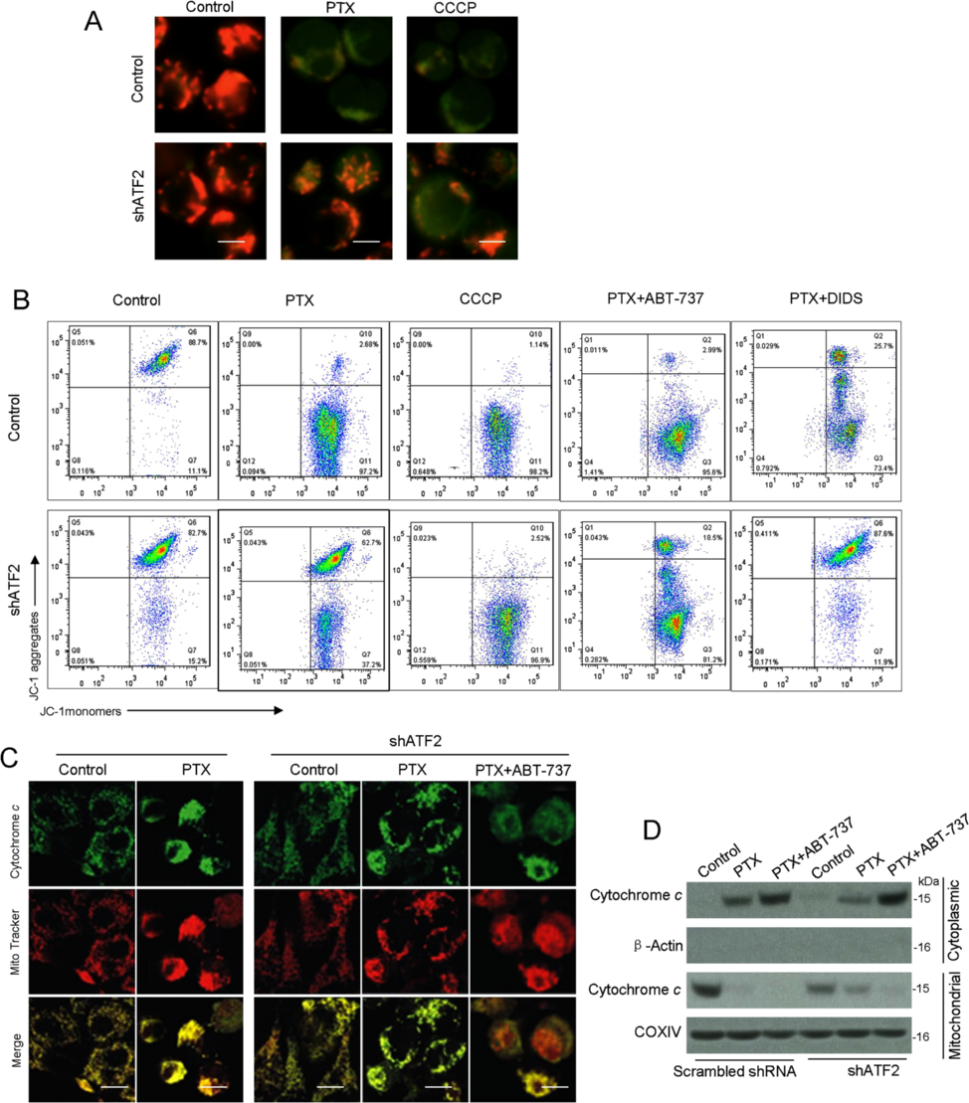
**ATF2 localization induces mitochondrial membrane damage, mitochondrial cytochrome** ***c*** **release, and caspase activation in B16F10 cells. (A)** B16F10 cells stably transfected with scrambled or ATF2 shRNA were incubated with either PTX or CCCP (50 M, 1 h). The cells were stained with JC-1 dye and observed the mitochondrial membrane potential by fluorescence microscopy. Green fluorescence indicates JC-1 stained mitochondrial plasma with a low JC-1 concentration (monomer) and depolarized mitochondrial membrane potential; red fluorescence indicates JC-1 stained mitochondrial plasma with a high JC-1 concentration (polymer)(Scale bar: 20 μm). **(B)** B16F10 cells stably transfected with scrambled or ATF2 shRNA were treated as indicated above. JC-1 flow cytometry analysis was performed and the represented diagrams of flow cytometry were presented. B16F10 cells stably transfected with scrambled or ATF2 shRNA were treated as indicated. The cells then were subjected to immunofluorescently stain with cytochrome *c* (Scale bar: 20 μm) **(C)** and immunoblot analysis for the mitochondrial and cytoplasmic extracts with the indicated antibodies **(D)**.

We next verify whether the caspase-dependent cell death can be extrapolated to canonical ATF2 mitochondrial localization. As indicated, cells expressing ATF2 shRNAi were exposed to PTX, and the mitochondrial membrane potential was analyzed to quantify JC-1 release. Consistent with previous reports, PTX caused a large, rapid decrease in the average red fluorescence signals compared to the controls, whereas the green fluorescence signals increased (Figure 5A). The ATF2 shRNAi-transfected B16F10 cells also did not exhibit alterations in either of the fluorescence signals in comparison with the cells transfected with the empty vector. In this setting, the fluorescence signals did not exhibit alterations following genotoxic stress compared to the control cells, when VDAC1 function was inhibited using DIDS, whereas the combination of PTX and ABT-737 caused increased green fluorescence signals in ATF2 depletion cells as efficiently as in control cells (Figure 5B). Thus, ABT-737 can restore the mitochondrial membrane potential alteration resulting from the loss of ATF2.

This observation was further corroborated by cytochrome *c* cytofluorometry and immunoblotting. The punctuate mitochondrial localization of cytochrome *c* was altered upon PTX to a diffuse cytoplasmic localization in the cells expressing native ATF2 (Figure 5C), which indicated that mitochondria cytochrome *c* had translocated to the cytosol. In contrast, the ATF2 depletion cells exibited a decreased intensity with diffuse staining (Figure 5C), which could be rescued by ABT-737, as evidenced by the release of cytochrome *c* into the cytoplasm. These results suggests that the apoptotic resistance of the ATF2 depletion cells is owing to the inability of cytochrome *c* release, further verified by western blotting to confirm the presence of cytoplasmic cytochrome *c* (Figure 5D). These findings clearly show that ATF2 is critical for cytochrome *c* release and suggest a link between mitochondrial ATF2 and cytochrome *c* release. Thus, ABT-737 can rescue the mitochondrial apoptotic response to PTX in B16F10 cells, indicating that ATF2 localization is able to activate the mitochondrial death pathway and trigger cell death in B16F10 cells through the cytochrome *c* release pathway.

### A decrease in ATF2 correlates with in vivo tumorigenicity

**Figure 6:**
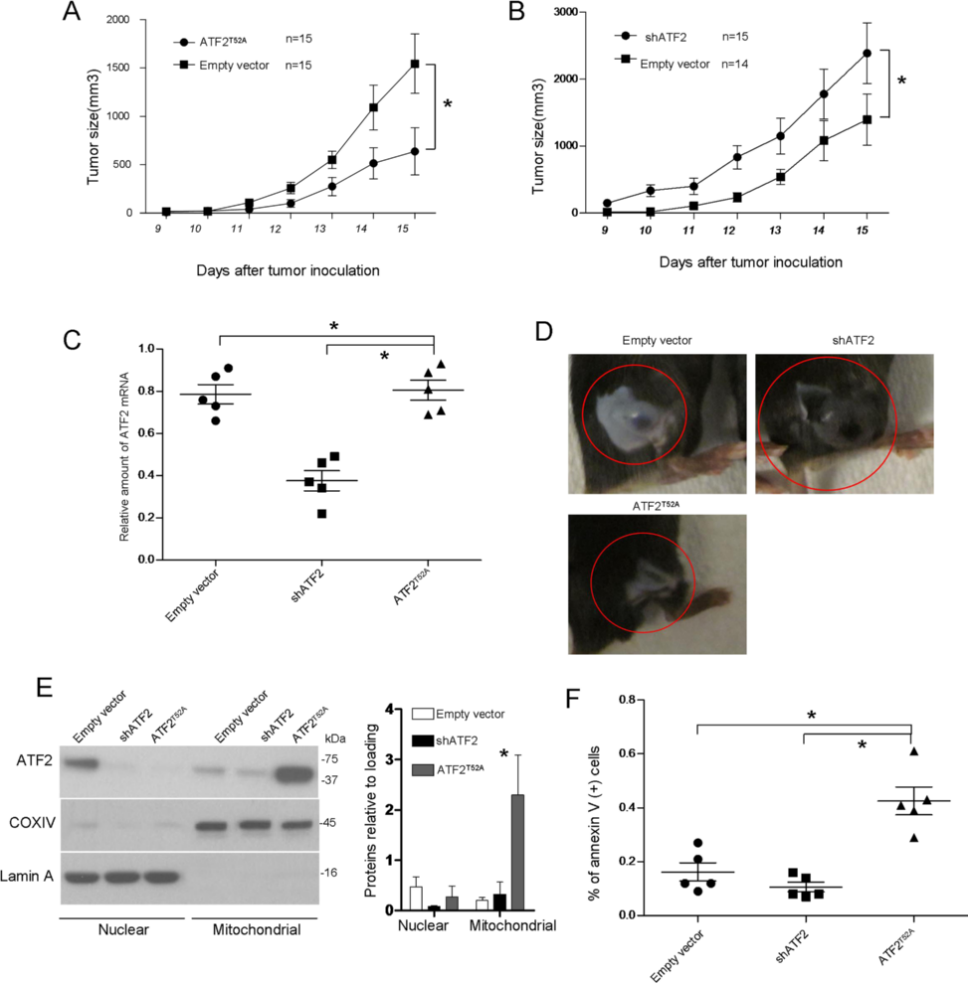
**Tumorigenicity of ATF2 in vivo.** B16F10 cells stably transfected with ATF2^T52A^ or ATF2 shRNA were injected into C57/BL6 mice and the tumor volume for ATF2^T52A^ tumor xenografts **(A)** and ATF2 shRNA tumor xenografts **(B)** was measured on the indicated day. Bars indicate s.e.m. *P < 0.01, Student’s t test. **(C)** The ATF2 mRNA levels in the cancer tissues isolated from tumor xenograft mice were measured by quantitative real-time RT-PCR (ANOVA, *P < 0.01, n = 6). **(D)** Representative pictures of mice bearing tumors at 3 weeks after B16F10 cells injection. **(E)** Mitochondrial and nuclear fractions were prepared from tumor xenografts and subjected to western blotting with an anti-ATF2 antibody at 3 weeks after B16F10 cells injection. The morphometry of ATF2 are indicated at right. The columns provide average values of at least 3 independent experiments performed in triplicate; the bars, SE. *P < 0.01 compared with the corresponding control. **(F)** After injection of the indicated tumor cells for 6 days, the xenografts were collected, and the in situ tumor cells were harvested and subjected to FACS apoptosis analyses by Annexin V/PI staining. Diagram presented quantitative analysis of three similar experiments. *P < 0.01, ANOVA. Data are means ± s.e.m.

We next examined the tumorigenic effect of ATF2 in the B16F10 cell line using RNA interference or mitochondrial ATF2 upregulation using ATF2^T52A^ transduction. ATF2 mRNA levels were significantly reduced in the ATF2-depleted xenograft tumors, slightly increased in the ATF2^T52A^ transduction xenograft tumors compare to the parental B16F10 empty vector control groups (Figure 6A). Tumor xenografts of B16F10 cells stably transfected with ATF2 shRNA exhibited a higher tumorigenic compared to the empty vector control (Figure 6B). As indicated in Figure 6B, the mean tumor volume of the B16F10-shATF2 group was significantly larger than the corresponding B16F10 groups (P < 0.05, One way ANOVA), whereas increased mitochondrial ATF2 is observed to block melanoma progression (Figure 6C, D). Similarly, the life span of the xenograft mice were also observed decrease in the B16F10-shATF2 cells and increase in the ATF2^T52A^ transduction B16F10 cells. Fractionation western blotting analysis using mitochondrial and nuclear fractions from mammary tumors indicated that the ATF2 protein was enhanced in the tumors from ATF2^T52A^ transduction cells and almost undetectable in the tumors derived from the ATF2-depleted cells (Figure 6E). Flow cytometry FACS analysis with annexin V and PI staining was used to determine cell apoptosis, with the early apoptotic cells in ATF2-depleted B16F10 melanoma cells being significantly lower compared to the empty vector-transfected B16F10 cells (Figure 6F), suggesting increased melanoma survival in the ATF2-depleted B16F10-induced tumor cells.

## Discussion

The cytosolic localization of ATF2 has been associated with tumor suppressor activity in some solid tumors [7,16-20]. However, targeting this pro-apoptotic protein for tumor treatment requires knowledge and understanding of their modes of action. Several possible mechanisms of action have been proposed in the previous reports. The present work offer insight into the mechanism by which mitochondrial ATF2 might help to cytotoxic stress induced mitochondrial membrane permeabilization (MMP). While the ATF2 still localizes to mitochondria and functions normally, releases of cytochrome *c* and apoptotic cell death were induced. Mitochondrial ATF2 impairs HK1/VDAC1 complexes and mitochondrial membrane integrity, sensitizing cells to apoptosis. Moreover, we demonstrated that apoptosis induction hierarchically involves Bim and VDAC1. The proapoptotic protein Bim was shown to promote apoptosis through interactions with VDAC1 and ATF2. Finally, a decrease in ATF2 mitochondrial expression led to enhanced tumorigenesis ability in vivo.

ATF2 appears to be a convergence point for a variety of cellular survival and death signals. Considering that the major cellular site targeted by mitochondrial ATF2 is the mitochondrion*,* several mechanisms were proposed to explain the action of such proteins at the mitochondria [1]. In accordance with previous results [3], genotoxic stress in melanoma cells was found to induce the predominant mitochondrial localization of ATF2 and promote cell death. The overexpression of mitochondrial ATF2 induced mitochondrial depolarization, a phenomenon associated with apoptosis, regardless of the cellular host. We also indicated that ATF2 depletion cells have a lower capacity to induce apoptosis in response to genotoxic stress, suggesting that lying in the mitochondrial outer membrane (MOM), ATF2 is a conserved mitochondrial element of the death machinery. These findings are consistent with previous results that the cytosolic ATF2 is necessary for mitochondria-mediated apoptosis (29). ATF2 is required for cytochrome *c* release when induced by various stimuli. Previous results indicated that the cytosolic ATF2 is necessary for mitochondria-mediated apoptosis [15], consistent with current findings. Cytochrome *c* release also found to ATF2 requirement with various stimuli exposure. Indeed, apoptosis induced of by PTX, cisplatin, and STS, acting on multiple pathways, all ultimately led to cytochrome *c* release in cells expressing native ATF2.

It has been shown that ATF2 might regulate and control the availability of VDAC1 to HK1, Bcl-2, and Bcl-xL [3]. Herein, we clearly show that ATF2 can interact with VDAC1 to disrupt the HK1/VDAC complex in various cancer cells, suggesting that ATF2 might bind to VDAC1 in competing with HK1 and thus promote cytochrome *c* release.

It has also been suggested that the proapoptotic Bim protein induces conformational changes in BAX to promote the targeting and homo-oligomerization of BAX at the MOM [21]. The proapoptotic proteins BAX and BAK were proposed to form heterodimeric complexes, with physical associations between VDAC1 and BAX being reported in various experimental models [22]. Here, we not only demonstrated a direct interaction of Bim with VDAC1 but also showed the function of this interaction in mediating the proapoptotic activity of ATF2 to modulate the interaction of VDAC1 and BAX. Our results point to a role for ATF2 as an activator of Bim. Serial siRNA experiments and epistatic analyses suggest that ATF2triggers the conformational activation of Bim, whereas, VDAC1 activation (downstream of ATF2 signal) need the Bim expression. Accordingly, VDAC1 does not adopt its active conformation when Bim is absent, whereas ATF2 remains inactive. Furthermore, pharmacological inhibition of Bcl-2/Bcl-X_L_ with the BH3 mimetic ABT-737, was able to rescue the defect of VDAC1 inactivation triggered by ATF2 deficient. It is worth noting that in the conditions used here, ABT-737 was not able to induce VDAC1 activation per se.

Therefore, our results define the pathway by which ATF2, Bim, and VDAC1 hierarchically operate together, which has not been reported to date. Although it is known that ATF2 can function upstream of Bim, at least in some paradigms of apoptosis, it has never been indicated that Bim might seat in a intermediate position between ATF2 and VDAC1, as present here. Furthermore, BAX and VDAC1 were reported to assemble amultimeric channel, large enough for the release of proteins [23-25]. Similarly, the biophysical interactions between Bim, ATF2, and VDAC1 might be important cytochrome c release. The requirement of ATF2 for cytochrome c release and its functional interaction with Bim indicate ATF2 as being critical factor in mediatting cell death. Future work must address the biochemical mechanisms underlying the cooperation between Bim and VDAC1. Interestingly, it has been shown that Bax and VDAC1 can form a large, multimeric channel that allows for the release of proteins. Hence, it will be important to investigate the putative physical and functional interactions between Bim, Bax and VDAC1 in the context of PTX-triggered cell death.

Concerning the function of ATF2 on tumorigenesis,we detected increased proliferation in tumors from an ATF2-deficient B16F10 cell line injected into C57BL/6 mice, in accordance with what occurs on apoptosis in ATF2-depletion cells. In fact, the mice that displayed an increased growth of B16F10 cells also showed a lower expression of ATF2 in the tumor tissue. This dominance has been detected in several other tumors [26,27]. Hence, we believe that the ATF2 may be more important in promoting tumor growth in this model, and it can be speculated that ATF2 might initially contribute to the events towards tumor proliferation.

## Conclusions

The current results bring light on the mechanism of mitochondrial ATF2 signaling via Bim and VDAC1 during cell apoptosis induced by PTX . Thus, ATF2 appear to be a promising apoptotic target for the therapeutic agents to work on, whereby targeting ATF2 to tumor cells suppressing proapoptotic proteins like Bcl-2 and HK1 would reduce the mechanisms for cancer cells self defense, accordingly, promoting cellular sensitivity to therapeutic ahents through increasing apoptosis. These observations provide full understanding of the mechanism role of ATF2 in modulation of antiangiogenic function, which might have implications in the future therapeutic agents design.
